# Epidemiology, clinical characteristics and life-threatening risk profile of WPW in children: a single-center experience in South Wales for 30 years

**DOI:** 10.1007/s00431-025-06252-z

**Published:** 2025-07-26

**Authors:** Orhan Uzun, Derya Duman, Gulhan Tunca Sahin, Yasemin Nuran Donmez, Afzal Abubakker Bapputty Haji, Mark Walsh, Cecilia Gonzalez Corcia, Peter O’Callaghan, Fong Leong, Alan Graham Stuart

**Affiliations:** 1 Department of Pediatric Cardiology, University Hospital of Wales, Heath Park, Cardiff, CF14 4XW Wales UK; 2 School of Medicine Cardiff University, Cardiff, UK; 3 School of Medicine Swansea University, Swansea, UK; 4 Royal Bristol Children’s Hospital, Bristol, UK; 5 Department of Cardiology, University Hospital of Wales, Cardiff, UK

**Keywords:** Wolff-Parkinson-White syndrome, Children, Atrial fibrillation, Sudden death

## Abstract

This study aims to assess the epidemiology, clinical presentation, management, and outcomes of pediatric Wolff-Parkinson-White (WPW) syndrome, as well as the incidence of life-threatening events (LTE) in South Wales. Retrospective review of patients (< 17 years old) diagnosed with WPW syndrome between 1986 and 2019 in South Wales. The study population consisted of 160 patients, of whom 86 (54%) were male. The prevalence of WPW syndrome was 0.028%. Thirty percent of cases were diagnosed during infancy, while 63% presented after age five. Incidental diagnosis occurred in 47 (29,3%) asymptomatic patients. Spontaneous resolution of delta wave in the entire cohort was observed in 19 patients (12%) while this phenomenon had occurred in 43% of neonates. Thirteen children [8.1% (13/160), 11 males] presented with serious arrhythmia giving an estimated risk of 3.8 per 1000 person-years during childhood in South Wales. Acute management was required in 36% (55/154) of patients, with seven (4.5%) experiencing pre-excited atrial fibrillation giving an estimated risk of 2 events per 1000 person-years. Among children with pre-excited atrial fibrillation and rapid ventricular conduction, only three out of seven children experienced aborted sudden cardiac death (1.9%) with an estimated life-threatening event risk of 1.7 per 1000 person-years. No deaths directly attributable to isolated WPW and related arrhythmia were recorded.

*Conclusions*: Asymptomatic WPW in children demonstrated a high propensity for life-threatening events in South Wales, prompting a policy shift towards earlier referral for electrophysiology study (EPS) before adolescence. These findings underscore the need for more rigorous risk stratification and closer follow-up of all WPW patients, as asymptomatic cases do not guarantee safety.

**Table:** 

**What’s new?** •*What is already known on this topic – Historically, asymptomatic patients with WPW and those demonstrating loss of preexcitation on exercise tests were considered low risk for life-threatening events (LTE) and managed conservatively by non-electrophysiologist.* •*What this study adds – This study reinforces the observation of a higher incidence of life-threatening events in asymptomatic children with atrial fibrillation and neonates with atrial flutter in the context of WPW and advocates for a more cautious approach, suggesting a lower threshold for referring younger and asymptomatic children for RF ablation procedures before adolescence.* •*How this study might affect research, practice, policy – The findings of this national study underscore the necessity for closer monitoring of infants and children with WPW syndrome, irrespective of age and symptoms. EPS emerges as the primary method for identifying individuals at high risk of LTEs during childhood.*

**Supplementary Information:**

The online version contains supplementary material available at 10.1007/s00431-025-06252-z.

## Introduction

Wolff-Parkinson-White (WPW) syndrome is characterized by accessory pathways connecting the atrium and the ventricle, which presents a risk of sudden arrhythmic death due to rapid conduction of atrial fibrillation or flutter to the ventricles, resulting in ventricular fibrillation [1]. Asymptomatic patients and those who present with loss of preexcitation on exercise testing have traditionally been considered at low risk for life-threatening events (LTE). However, emerging evidence challenges this perception, suggesting a potential underestimation of risk, particularly in younger populations [2, 3].

Yet, no population-based study in the United Kingdom (UK) has comprehensively investigated the epidemiology, clinical presentation, and risk of life-threatening events associated with WPW syndrome. This is the first study of its kind in the UK. Therefore, this study aims to fill this gap by evaluating these aspects among pediatric WPW patients at a tertiary cardiac center in South Wales.

## Methods

This is a retrospective review of all patients diagnosed and treated at the National Children’s Hospital for Wales over the past 30 years. The paediatric cardiology department provides tertiary pediatric cardiology services for a population of 2.5 million in South Wales. Surgical and interventional procedures for children with cardiac problems from Wales are provided at Bristol Children’s Hospital which is the designated center for the South Wales and Southwest of England congenital cardiac network. University Hospital of Wales receives all pediatric cardiac referrals from eight district general hospitals in South Wales. All pediatric patients from fetus to age 18 are referred to, managed, and followed up by a pediatric cardiologist in the tertiary pediatric cardiology unit at the Children’s Hospital for Wales. Whether they are symptomatic or not, all children with an abnormal electrocardiography (ECG) including preexcitation are recorded in the departmental and national database “Cardiobase.” This study was conducted on 160 patients under 17 years old diagnosed with WPW syndrome between 1986 and 2019 in South Wales. Patient data were sourced from hospital medical records and departmental digital databases. Collected data encompassed patient demographics, presence of associated heart disease, clinical presentation, documented arrhythmias, persistent or intermittent character of preexcitation, spontaneous resolution of manifest preexcitation, findings of invasive electrophysiology studies (EPS), and clinical events during follow-up. Only small PDA or PFO were excluded from the definition of congenital heart disease. The ECG criteria of atrial and ventricular enlargement/hypertrophy and abnormal QRS electric axis deviation are interpreted according to the European and American Society of Cardiology guidelines [4, 5].

Asymptomatic patients were described as those who had evidence of preexcitation on the ECG but no documented arrhythmia or specific symptom such as palpitation indirectly indicating SVT. The presence of AP was documented incidentally when they were examined for other reasons, such as murmur, seizures, or sports and family screening. Symptomatic patients were classified as those who had palpitations associated with or without chest pain, dyspnea, fainting, and exercise limitation. Neonates, infants, and small children can not describe palpitation and the usual presentation of SVT in infants occurs with non-specific symptoms such as poor feeding, agitation, signs of cardiovascular compromise. Therefore, patients in these age groups presenting with such findings were considered symptomatic.

Non-invasive risk stratification included Holter monitoring and/or exercise stress testing to demonstrate loss of ventricular preexcitation as described before [6]. Holter was analyzed by two electrophysiologists, and the findings were confirmed either with clinical tachycardia requiring adenosine or during the EPS. All children undergoing exercise tests and Holter monitoring met the endpoint of achieving more than 85% of age-predicted heart rate. The abrupt disappearance of preexcitation during exercise was considered to be a lower risk by conventional criteria. Persistence and gradual lessening of preexcitation both were regarded in the same high-risk group. EPS data comprised accessory pathway (AP) location(s), AP conduction properties, and inducibility of tachycardia. High-risk APs were defined as having specific criteria for antegrade AP effective refractory period (APERP), shortest pre-excited paced cycle length (SPPCL) during atrial pacing or shortest pre-excited RR interval in atrial fibrillation (SPERRI) ≤ 250 ms at invasive EPS [2, 7]. EPS is performed under general anaesthetics in all children below 16 years of age at our institution. Isoproterenol (isoprenaline) was not routinely used, but it was only given in selected cases (if there was any benefit) to reproduce sustained atrial fibrillation. Atrial and ventricular stimulation during the EP study was continued until ERP of the AV node was reached or the accessory pathway block had occurred. The intensity of the stimulation did not intend to reproduce atrial fibrillation specifically unless AF had occurred spontaneously before the AV node or accessory pathway effective refractory period had been reached. Atria were stimulated at a cycle length of 300 ms by a pacing train of eight beats followed by a single or double premature stimulus being delivered at progressively shortening coupling intervals of 10ms decrements until an arrhythmia was induced, or the first extra stimulus had lost capture, with the shortest coupling interval being at 180 ms. Ablation outcomes and complications were documented. Non-persistent preexcitation was defined as intermittent absence of ventricular preexcitation on ECG or Holter monitoring or sudden loss of delta wave during exercise stress testing.

Spontaneous resolution is based not only on spot ECG, but also on 24-Hour Holter in all patients (instructed to undertake running and brisk walking exercise activity while wearing the monitor) and in selected patients a treadmill exercise test performed when the age was appropriate. The Holter was repeated on two occasions between 6 and 12 months apart before concluding spontaneous resolution [6, 8].

Life-threatening events (LTEs) were classified as sudden death or aborted sudden death. Life-threatening arrhythmias (LTA) represent clinical episodes of pre-excited atrial fibrillation associated with or without hemodynamic compromise, syncope, or seizure. All these arrhythmias, which could cause a life-threatening situation and/or need an intervention, are referred to as serious arrhythmias (SA). Atrial flutter (AFl) and double tachycardia (atrioventricular reentrant tachycardia—AVRT and AFl), causing hemodynamic compromise and necessitating intubation for acute direct current (DC) cardioversion, were also considered SA as manifestations of WPW syndrome [2, 9–13]. LTE events are considered in two distinct categories: first, the traditionally accepted risk of pre-excited atrial fibrillation leading to VF, and the second, atrial flutter leading to fast ventricular rates, hemodynamic collapse, and shock as the new risk factor proposed by this study. Data were tabulated, grouped, and analyzed using descriptive statistics.

The population prevalence of WPW syndrome was determined by dividing the number of subjects in the study cohort by the total number of subjects aged 0–18 years in the population over the study period in South Wales.

## Statistic methods

Data analysis was performed using IBM SPSS Statistics 22 (IBM SPSS, UK). The normality of parameters was assessed using the Shapiro–Wilk test. Descriptive statistics (mean, standard deviation, frequency) were employed for data evaluation. For comparisons of normally distributed quantitative data between two groups, the Student *t*-test was used, while the Mann–Whitney *U* test was applied for non-normally distributed parameters. The Chi-square test, Fisher’s Exact test, Fisher Freeman Halton test, and Continuity (Yates) Correction were utilized for qualitative data comparisons. Multivariate analysis was conducted using logistic regression analysis. Survival time comparisons among age groups were performed using the Log-Rank (Mantel-Cox) test, with the impact of age groups on survival further investigated through Cox regression analysis. A level of significance was considered if the *p* was less than 0.05.

## Ethics standards

As the data were collected and recorded as part of routine clinical practice for periodic service evaluation, a specific ethical approval application was deemed unnecessary. Both the Health Research Authority of the UK and the South Wales Ethics Committee confirmed that reviews of this nature do not require ethics approval. Before retrospective analysis, all collected information was anonymized. Additionally, all visiting research fellows (DD, GTS, YND) as co-authors held official contracts with the University Hospital of Wales during the review and analysis period. All procedures about this review adhered to the principles outlined in the Declaration of Helsinki.

## Results

### Demographic and clinical data

**Fig. 1:**
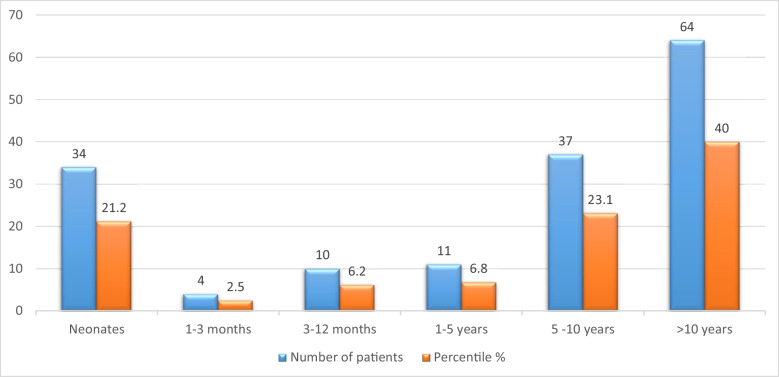
Age at presentation of patients with WPW syndrome

Over a 30-year period, 160 patients were included in the study, with 54% being male. The median age at diagnosis was 8 years (range 0 to 16 years). The patients’ follow-up duration was a median of 7 years (2–25 years). The childhood prevalence of WPW syndrome was 0.028% [160/562,730 (total population of children below 17 years of age during the study period)]. Diagnosis of WPW was made during infancy in 30% of cases, in 21% during neonatal period and 63% patients were diagnosed after 5 years of age (Fig. 1). Incidental diagnosis of delta wave was noted in 29% ( *n *= 47) of asymptomatic patients. The most common presenting symptoms in older children were palpitations at rest (74/160: 46%) and on exertion (25/160: 16%), followed by dizziness/lightheadedness (34/160: 21% ) and shortness of breath (19/160: 12%). Neonates and infants presented with less specific symptoms such as poor feeding, agitation, and signs of cardiorespiratory compromise.

Holter ECG was obtained in 52% of patients (*n* = 83), with supraventricular tachycardia (SVT) being captured in 18% of them. Exercise stress tests were performed in 32.5% of patients (*n* = 52), with the abrupt disappearance of delta wave being noted in 29% (*n* = 15) of the subjects. Significant structural heart disease was detected in nine patients, including Ebstein’s anomaly (*n* = 3), hypertrophic cardiomyopathy (*n *= 2), Fallot’s tetralogy, ventricular septal defect with pulmonary stenosis, total anomalous pulmonary venous connection, and sinus venosus type atrial septal defect with pulmonary stenosis (for each, *n* = 1). Left ventricular (LV) systolic function was impaired in 13% of patients (*n* = 21), of which 11 were infants and three neonates. Moreover, two patients had hypertrophic cardiomyopathy, one due to Noonan and other Danon syndromes.

**Fig. 2:**
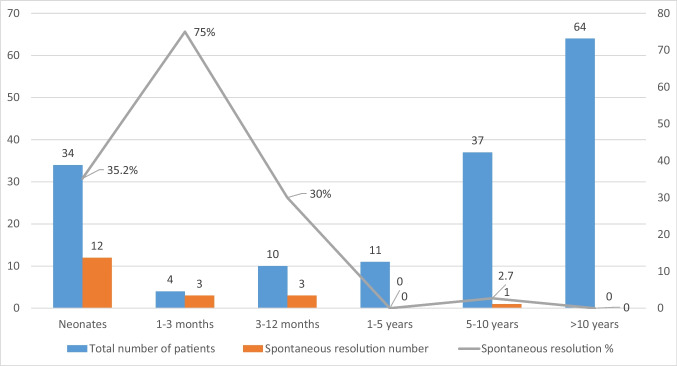
Spontaneous resolution of delta wave (*n* = 19) according to the age groups at diagnosis

Spontaneous resolution of delta wave was observed in 12% of all patients (*n* = 19), with only 8.5% of asymptomatic patients experiencing resolution. However, spontaneous resolution rates were higher at 43% and 35% in neonates (<28 days) and infants (<1 year) respectively (Table 3). 24-Hour Holter monitoring and/or exercise stress testing were carried out for these patients. The latest spontaneous resolution of delta wave was noted to have occurred at 17 years of age. The age distribution at which spontaneous resolution of delta wave had occurred is shown in Fig. 2.

Full data on acute management was available only in 154 patients. Acute management according to the Advanced Paediatric Life Support (APLS) protocol was required in 36% of patients (*n* = 55), with adenosine being administered to 69% of them (*n* = 38). Among those who received adenosine, 66% (*n* = 25) responded to treatment, while 8% (*n* = 13) required cardioversion, and the remaining had further intravenous antiarrhythmic treatment.

Patients who were not initially diagnosed in our center and did not attend regular follow-ups were excluded from the study.

### Electrophysiology study data

Table 1 presents EPS data and ablation outcomes. Some of the conduction properties showed that there were anatomically distinct multiple accessory pathways. Of the 101 patients (63%) who underwent EPS and ablation procedures, 18 (18%) had multiple accessory pathways (APs), with a slight majority located on the right side (52.5%). Notably, ablation of single right APs (27%) failed twice as often as that of single left-sided ones (13.5%).

**Table 1:** Baseline EPS data and ablation outcomes

		Asymptomatic, ***n*** (%)	Symptomatic, ***n*** (%)	Total, ***n*** (%)	***p***
Gender (male)		28 (%59.6)	58 (%51.3)	86 (%53.8)	^2^0.341
Age groups	< 1 month	7 (%14.9)	27 (%23.9)	34 (%21.3)	^3^0,023*
	1 month–1 year	9 (%19.1)	5 (%4.4)	14 (%8.8)	
	1–5 years	4 (%8.5)	7 (%6.2)	11 (%6.9)	
	> 5 years	27 (%57.4)	74 (%65.5)	101 (%63.1)	
EPS performed		21 (%44.7)	80 (%70.8)	101 (%63.1)	^4^0.002*
Ablation performed		17 (%36.2)	79 (%69.9)	96 (%60)	^5^0.000*
APERP (ms) (***n*** = 60) (Min–Max)–(Mean ± SD)		(220–450)–(300 ± 62.82)	(180–440)–(297.73 ± 51.75)	(180–450)–(298.33 ± 54.37)	^1^0.888
SPERRI (ms) (***n*** = 60) (Min–Max)–(Mean ± SD)		(220–450)–(303.13 ± 64.05)	(180–400)–(295 ± 47.03)	(180–450)–(297.17 ± 51.65)	^1^0.594
Risk stratification		***n* (%)**	***n* (%)**	***n* (%)**	
Serious arrhythmias		1 (%2.1)	12 (%10.6)	13 (%8.1)	^2^0.062
APERP (≤ 250 ms)		13 (%81.3)	35 (%79.5)	48 (%80)	^2^0.599
SPERRI (≤ 250 ms)		13 (%81.3)	35 (%79.5)	48 (%80)	^2^0.599
SVT (ORT) induced		7 (%33.3)	32 (%40)	39 (%38.6)	^3^0.759
SVT (ART) induced		1 (%4.8)	0 (%0)	1 (%1)	^2^0.208
> 1 accessory pathway		3 (%14.3)	15 (%18.8)	18 (%17.8)	^2^0.455
Ablation successful		14 (%61.9)	67 (%71.3)	81 (%84.4)	^3^0.575
Procedure complication		1 (%4.8)	4 (%5)	5 (%5)	^2^0.723
Bronchospasm due to adenosine		1 (%100)	0 (%0)	1 (%25)	
Complete AV block		0 (%0)	1 (%33.3)	1 (%25)	
Pericardial laceration		0 (%0)	1 (%33.3)	1 (%25)	
Pericardial puncture		0 (%0)	1 (%33,3)	1 (%25)	

*APERP* accessory pathway effective refractory period, *ART* antidromic reciprocating tachycardia, *EPS* electrophysiology study, *ORT* orthodromic reciprocating tachycardia, *SPERRI* shortest pre-excited RR interval in atrial fibrillation, *SVT* supraventricular tachycardia

^*^p < 0.05

^1^Student *t*-test

^2^Fisher’s exact test

^3^Continuity (Yates) correction

^4^Chi-square test

^5^Fisher Freeman Halton Test

Among patients with multiple APs (*n* = 14), 78% had a failed initial attempt of ablation. Moreover, four patients with postero-septal pathways and one with a pathway in the middle cardiac vein also had a failed first ablation. Sixty-seven percent (*n* = 22) of patients with failed ablations underwent repeat EPS, with 18.2% (*n* = 4) still experiencing unsuccessful repeat ablations. The overall success rate with EPS, including repeat ablations, was 84.4% in the context of three different electrophysiologists’ learning curves during three different eras.

Failure is defined as the persistence of preexcitation at the end of the EPS. The variables associated with a higher failure rate with ablation included multiple APs 77.8% (versus 22.9% with single AP), right-sided single APs 27% (versus 13.5% left sided pathways), and pathways located within the posteroseptal region 80% or middle cardiac vein 100%.

Comparison of EPS data between symptomatic and asymptomatic groups revealed no significant differences in mean APERP, SPERRI, or SPPCL values (Table 1). Similarly, there were no differences in the proportions of both groups with APERP, SPERRI values of ≤ 250 ms, multiple accessory pathways, procedural success rate, or complications. The rate of potentially dangerous pathways with short APERP and SPERRI (≤ 250 ms) was similar in symptomatic and asymptomatic patients (*p* = 0.599). EPS identified right APs in seven patients, left APs in five patients, more than one AP in six patients, and para-Hisian AP in two asymptomatic patients. Table 1 summarizes the AP characteristics of both asymptomatic and symptomatic patients.

### Outcomes

Thirteen children (8%, 84.6% male) experienced serious arrhythmias (SA) with five ≤ one month old. The total SA frequency was 3.8 events per 1000 person-years in the South Wales child population. Seven patients required medical attention due to pre-excited atrial fibrillation (AF), resulting in a pre-excited AF (LTE) risk rate of 2 events per 1000 person-years. Among patients with pre-excited AF and rapid ventricular conduction, three experienced aborted sudden cardiac death (SCD) (1.9%). These three asymptomatic children had their first presentation with pre-excited AF degenerating into ventricular fibrillation (VF) and required cardiopulmonary resuscitation (CPR), with a (LTE) risk rate of 1.7 per 1000 person-years. LTE predominantly occurred at rest or with non-competitive activity (*n* = 11, 84.6%). Table 2 outlines case subject characteristics and LTE details, with SA being the first presentation in 12 cases, none of whom had previous symptoms or were known to have WPW. Although SA had occurred in symptomatic patients 5 times higher (10.6%) than asymptomatic ones (2.1%), the difference was not found to be statistically significant (*p* = 0062). In patients with SA, high-rate tachycardia above 250 beats/min was present, causing heart failure and shock. These patients also had double tachycardia with AVRT. Amongst 13 patients with serious arrhythmia, six cases presented with double tachycardia involving atrial flutter and AVRT, and seven presented with pre-excited atrial fibrillation degenerating into VF due to rapid conduction. All three patients who experienced aborted SCD (cases 6,7, and 8 as in Table 2) and cardiovascular collapse necessitating resuscitation and defibrillation, had one left lateral AP. However, the two older children, the case 7 had one more, and the case 8 had two more additional right-sided accessory pathways.

**Table 2:** Presentation, management, and outcome of serious arrhythmias, representing life-threatening arrhythmias and events in WPW patients

No	Age	Sex M/F	Clinical presentation	Symptoms at presentation	Activity	Abnormal ECG before LTE**	Treatment	Associated structural/functional heart disease	Accessory pathway(s)	Outcome
1	1 day	M	Atrial flutter, AVRT	Hemodynamic collapse	Non-competitive (rest)	First presentation	DC cardioversion, medical treatment	Severe LV dysfunction	–	ECG WPW, awaiting EPS
2	1 day	M	Atrial flutter, AVRT	Hemodynamic collapse	Non-competitive (rest)	First presentation	DC cardioversion, medical treatment	Severe LV dysfunction	Left lateral	Successful ablation on 1st attempt
3	3 weeks	F	Atrial flutter, AVRT	Hemodynamic collapse	Non-competitive (rest)	First presentation	DC cardioversion, medical treatment	–	–	Controlled off medication (sotalol)
4	1 month	M	Atrial flutter, AVRT	Hemodynamic collapse	Non-competitive (rest)	First presentation	DC cardioversion, medical treatment	Moderate LV dysfunction	–	Spontaneous resolution, one-year follow-up
5	1 month	M	Atrial flutter, AVRT	Hemodynamic collapse	Non-competitive (rest)	First presentation	DC cardioversion, medical treatment	–	–	Spontaneous resolution, one-year follow-up
6	2 months	M	Atrial flutter, AVRT	Hemodynamic collapse	Non-competitive (rest)	First presentation	CPR, DC cardioversion	LV dysfunction	Left anterolateral	Successful ablation on 1st attempt
7	5 years	M	Pre-excited AF to VF epilepsy, LOC	Aborted sudden cardiac death	Non-competitive (rest)	Asymptomatic WPW	CPR, defibrillation	–	Multiple pathways (left sided coronary sinus mouth + right posterior)	Successful ablation on 2nd attempt
8	7 years	M	Pre-excited AF to VF	Aborted sudden cardiac death	Competitive (football)	First presentation	CPR, defibrillation	–	Multiple AP left sided	Successful ablation on 1st attempt
9	9 years	M	Pre-excited AF to VF	Chest pain palpitation syncope	Non-competitive (walking)	First presentation	Spontaneous recovery with medical treatment	–	Right posteroseptal	Successful ablation on 2nd attempt
10	14 years	M	Pre-excited AF to VF	Aborted sudden cardiac death	Competitive (football)	First presentation	Defibrillation medical treatment	–	Left sided coronary sinus diverticulum	Successful ablation on 1st attempt
11	15 years	M	Pre-excited AF to VF partial seizure	Palpitation, seizure, syncope	Non-competitive	First presentation	Spontaneous recovery with medical treatment	–	Parahisian	Successful ablation on 1st attempt
12	15 years	M	Pre-excited AF to VF	Palpitation, dizziness, pre-syncope	Non-competitive	First presentation	Spontaneous recovery with medical treatment	–	Right anterolateral	Successful ablation on 2nd attempt
13	15 years	F	Pre-excited AF to VF	Palpitation, syncope	Non-competitive	First presentation	Spontaneous recovery with medical treatment	–	Left lateral, left mid-septal	Successful ablation on 2nd attempt

*AF* atrial fibrillation, *AP* accessory pathway, *AVRT* atrioventricular reantrant tachycardia, *CPR* cardiopulmonary resuscitation, *DC* direct current, *ECG* electrocardiography, *EPS* electrophysiological study, *F* female, *LOC* loss of consciousness, *LTE* life-threatining event, *LV* left ventricle, *M* male, *SVT* supraventricular tachycardia, *VF* ventricular fibrillation, *VT* ventricular tachycardia, *WPW* Wolff-Parkinson-White syndrome

**Fig. 3:**
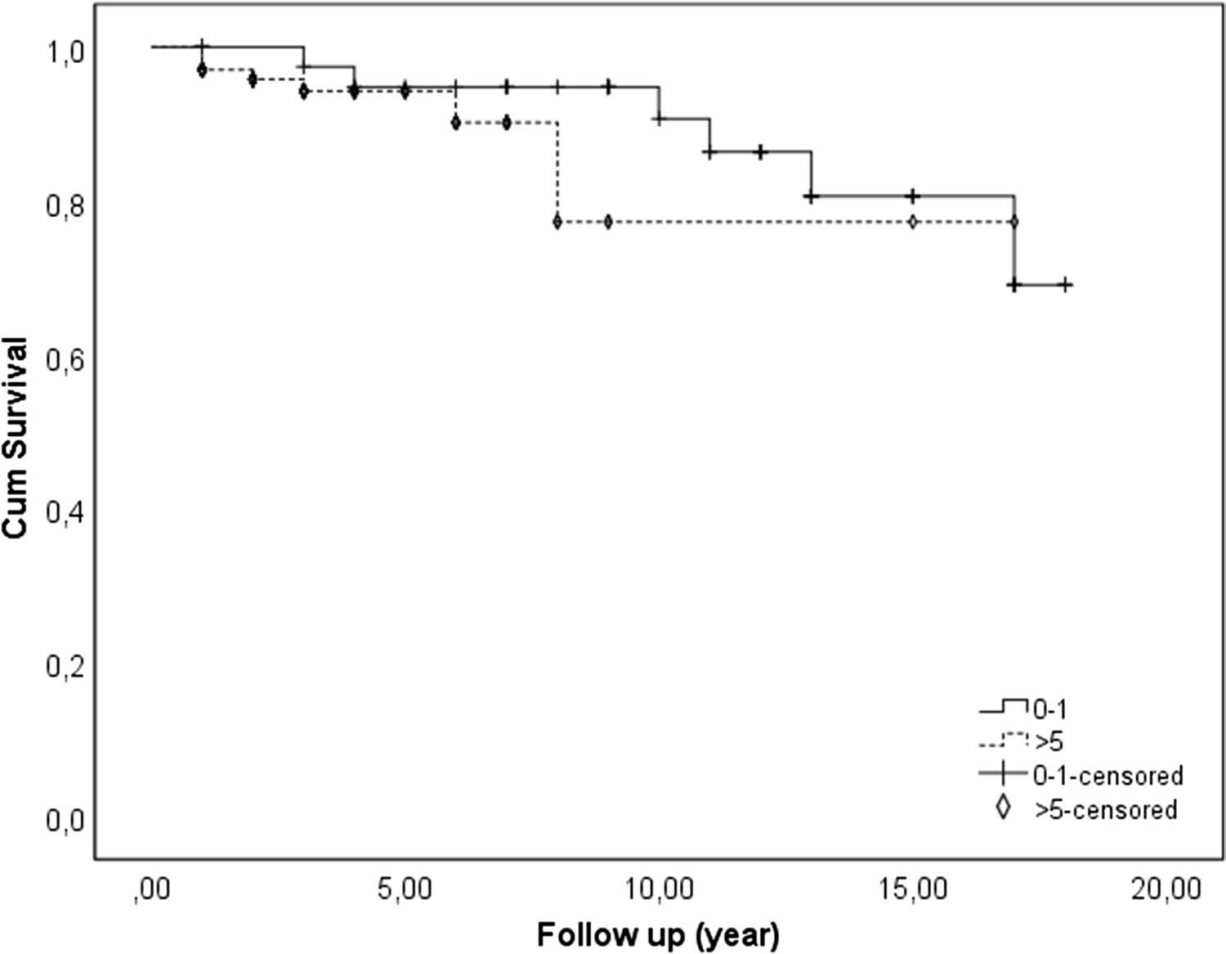
Survival curve of patients with WPW syndrome

When comparing SA survival time across age groups, no significant difference was found (p = 0.339). However, patients in the > 5 years age group had a -1.9 times higher risk of sudden cardiac death compared to the 0–1 year age group, although not statistically significant (*p* = 0.343) (Fig. 3). Notably, six patients presenting with pre-excited AF were in the > 5 years age group. Table 3 summarizes and compares findings among neonates, infants, and others.

**Table 3:** Characteristics of the patients with WPW syndrome according to the age groups

	Neonates ***n***: 34	Infants (< 1 year old) ***n***: 14	Children (> 1 year old) ***n***: 112
First presentation SA, *n*	3	3	7
First presentation LTE, *n*	–	1	2
Asymptomatic patients, *n*	6	12	29
Pre-excited AF,* n*	0	0	7
Decreased LV function, *n*	3	11	7
Spontaneous resolution, *n*	12	6	1

*AF* atrial fibrillation, *LTE* life-threatening event, *N* number, *WPW* Wolff-Parkinson-White Syndrome, *SA* serious arrhythmias

Complete clinical recovery occurred in all cases of cardiac failure except for one subject who experienced acute kidney failure post-SA, necessitating kidney transplantation in his teenage years. No deaths attributable to WPW or related arrhythmias were recorded in this cohort. However, one patient with preexcitation and documented arrhythmia from neonatal to teenage years died following heart transplantation due to severe myocardial failure secondary to Danon disease.

## Discussion

This study represents the largest epidemiological investigation of children with WPW syndrome in the UK, offering valuable insights into prevalence and LTE risk. Our findings show a WPW prevalence of 0.028% among children in South Wales, aligning closely with similar studies globally [3]. In this study, the total SA frequency is 3.8 events per 1000 person-years and pre-excited AF leading to rapid ventricular conduction risk rate is 2 events per 1000 person-years. Notably, 1.9% of children presented with aborted sudden cardiac death, indicating a considerable LTE risk of 1.7 events per 1000 patient-years, consistent with prior research [3, 8, 14, 15]. Undoubtedly, the true incidence of WPW among children may be underestimated, considering asymptomatic cases and undocumented sudden cardiac deaths. However, these omissions are unlikely to significantly alter prevalence estimation in our cohort. We observed a higher LTA risk in males (ratio of 5 to 1), consistent with existing literature, despite comparable preexcitation occurrence in both sexes [14].

Historically, asymptomatic WPW is considered benign, but emerging evidence challenges this notion. Our study corroborates previous findings that LTE risk in asymptomatic patients is similar to symptomatic patients [16]. In this regard, Sarubbi et al. [17] reported that 30% of initially asymptomatic individuals had developed symptoms over a 12-year follow-up period. A recent meta-analysis reported the overall risk of SCD for asymptomatic WPW patients at 0.85 events per 1000 person-years. It should be emphasized here that out-of-hospital mortality was not represented in their analysis, which can explain the lower rate in their study compared to ours. Even our study may not have captured all out-of-hospital arrests with undiagnosed WPW. Therefore, the true risk for sudden cardiac death for asymptomatic WPW could have been higher than what we have reported here, despite being the only tertiary pediatric cardiac center in South Wales.

While strenuous activities are commonly associated with an elevated LTE risk, it is noteworthy that sports restrictions alone would not have averted all LTE occurrences. A recent study revealed that 73% of LTAs did not occur during any competitive activity [2], aligning with our findings where the majority of LTAs (85%) occurred without competitive sportif engagement. Even, adolescents with pre-excited atrial fibrillation and rapid ventricular conduction at rest can be conscious and ambulatory with complaints of fatigue, dizziness and palpitation as it had happened in one case among this cohort (Supplementary Material). Nonetheless, this typical arrhythmia requires emergency care and rapid action with mostly DC cardioversion regardless of patient's symptoms as the arrhythmia can lead to hemodynamic compromise and loss of consciousness very quickly. These observations echo previous reports highlighting that children with preexcitation are susceptible to LTEs even during periods of rest or minimal activity[18–20].

We have previously shown that as much as 25% of fetuses and neonates with atrial flutter display evidence of preexcitation and develop a second type of atrioventricular tachycardia antenatally or postnatally [11]. Our cohort also revealed some instances where atrial flutter in neonates with WPW led to cardiovascular collapse, emphasizing the severity of such events. Consequently, we advocate for including pre-excited atrial flutter in the definition of LTA among neonates with WPW.

A noticeable case in our study involved a 5-year-old boy with asymptomatic preexcitation who suffered an episode of aborted SCD due to rapid conduction of AF during follow-up, necessitating CPR with defibrillation. Remarkably, this child was also known to have epileptic seizures, raising the possibility that these seizures could have been triggered by unrecognized arrhythmic events. This case underscores the critical importance of meticulously evaluating electrocardiograms (ECGs) in children with unexplained or atypical epileptic seizures.

It is also well known that ventricular preexcitation can have detrimental effects on systolic function over time due to abnormal depolarization and repolarization of the myocardium. Accessory pathways can disrupt the typical sequence of ventricular activation, potentially leading to ventricular dyssynchrony, with a higher impact on the right free wall and posterior septum locations [21]. Studies have demonstrated that ablation of these accessory pathways can restore normal ventricular function during follow-up [22–24]. Consistent with these findings, our study observed that impaired left ventricular systolic function in all 18 patients (11.2%) returned to normal ranges following ablation.

In tis study spontaneous resolution rates of delta wave were higher at 43% and 35% in neonates (<28 days) and infants (<1 year) respectively as compared to <1% in children beyond one year of age (see Table 3). Even if spontaneous resolution of WPW is possible (10-35%) in infants, it is only observed in the minority of patients beyond the first year of life [8]. Moreover, it remains crucial to continue with follow up of these patients who have ostensibly resolved delta wave, as preexcitation can re-emerge in 30% of such cases around the age of 8 years [25]. In our study, preexcitation re-emerged 12 years after diagnosis in one case. In another case, there was no evidence of a delta wave on 12-lead ECG, Holter, and exercise tests throughout childhood, but preexcitation manifested at 25 years of age with SVT. Therefore, non-invasive risk stratifications of WPW syndrome in children with those aforementioned methods alone cannot be relied upon either. In such symptomatic patients EPS should be a preferred approach.

The utilization of EPS to assess the risk associated with accessory pathways has traditionally centered on parameters like APERP, shortest pre-excited R-R interval during atrial pacing (SPERRI-Ap), and shortest pre-excited R-R interval during atrial fibrillation (SPERRI-AF) [26]. However, it is increasingly evident that there are no definitive cutoffs in these non-invasive/invasive risk markers that guarantee 100% sensitivity. Consequently, patients with accessory pathways deemed low-risk based on benign conduction properties measured during electrophysiology studies may still experience life-threatening events (LTEs) [2, 26, 27]. A recent large multicenter study underscored this concern, revealing that 37% of children with LTEs in the series lacked high-risk accessory pathway characteristics during electrophysiology studies [2].

Moreover, when risk stratification was conducted, a study found that clinical SPERRI and SPERRI at EPS exhibited only moderate correlation under anesthesia, with 76.1% of asymptomatic and 55% of symptomatic patients showing notable discrepancy [2]. In addition, Etheridge et al. demonstrated that a quarter of patients with a clinically high-risk SPERRI would have been erroneously classified as low-risk [2, 26]. Our findings align with these conclusions, as we observed no significant differences in mean APERP, SPERRI, and SPPCL values between patients with multiple accessory pathways and those with asymptomatic preexcitation. The occurrence rate of potentially hazardous pathways with short APERP was comparable between symptomatic and asymptomatic patients too. Yet another study about risk analysis for arrhythmia of non-persistent preexcitation in children with WPW described a high-risk AP and/or SCD or pre-excited atrial fibrillation in some cases [7].

The majority of patients in our cohort underwent radiofrequency ablation, with an overall success rate of the initial procedure at 84.4%, which may appear lower than contemporary results. However, our cohort includes patients from three different periods and three different electrophysiologists’ practices. After repeat ablations, a success rate of 100% was achieved, reflecting WPW as a condition largely amenable to cure. Left-sided accessory pathways were more common in patients experiencing LTEs. Notably, four patients required a repeat third ablation procedure to achieve successful eradication of the accessory pathways which is in keeping with the previous reports [2, 26].

An important consideration in the ablation treatment of asymptomatic WPW cases is the risk of procedural complications, particularly in paediatric patients. However, a recent study investigating the safety of paediatric ablation in the UK revealed negligible occurrence rates of major complications such as heart block (12/7069; <0.01%) or neurological injury [28]. Therefore, electrophysiology studies and RF ablation procedures may be considered in children over 6 years of age with a weight of more than 25-30 kg for investigating and treating APs associated with WPW syndrome regardless of symptoms.

## Conclusions

This study underscores a heightened risk of serious arrhythmias and life-threatening events in asymptomatic children as well as in infants with WPW syndrome due to pre-excited atrial flutter in addition to atrial fibrillation in older children in South Wales. Consequently, the findings of this national study emphasize the need for more vigilant monitoring of infants and young children with WPW, irrespective of age or symptoms. Moreover, our study suggests a more proactive approach to referring younger children for radiofrequency ablation procedures before adolescence. Future epidemiological studies should focus on establishing safer investigative methods, more reliable risk stratification techniques, and robust follow-up protocols. Given the unreliability of non-invasive risk stratification with exercise test in WPW patients, electrophysiology study emerges as the primary mechanism for identifying high-risk individuals for life-threatening events during childhood.

## Study limitations

While this study benefits from a robust central database for children in South Wales, its retrospective nature inherently suffers from unavoidable limitations. One notable constraint is the inability to capture clinically silent and undiagnosed cases of WPW syndrome, potentially resulting in the underrepresentation of the true prevalence. Additionally, the incidence of sudden death or cardiac arrest may have been underestimated due to the unavailability of electrocardiography in such cases. However, we believe that the number of unrecognized WPW cases was likely to be minimal. Our unit oversees all children with incidentally detected arrhythmia in all 8 district general hospitals whether a pre-excitation pattern was seen during sports screening or when a child was investigated for any other systemic illnesses.

## Supplementary Information

Below is the link to the electronic supplementary material.

ESM 1 (DOCX 2.21 MB)

ESM 2 (JPG 4.62 MB)

## Data Availability

The data that support the findings of this study are not openly available due to reasons of sensitivity and are available from the corresponding author upon reasonable request.
