# Supplementary material for: Epidemiology, clinical characteristics and life-threatening risk profile of WPW in children: a single-center experience in South Wales for 30 years
Source: Eur J Pediatr. 2025 Jul 26;184(8):504. doi: 10.1007/s00431-025-06252-z (PMC12296802; doi:10.1007/s00431-025-06252-z)

**Supplementary Material.** This 15 year old patient presented to a nearest general  hospital with complaints of palpitation, dizziness and feeling unwell and faint. (a) ECG performed at the adult coronary care unit showed pre-excited atrial fibrillation with rapid ventricular conduction. There was no loss of consciousness, but observation revealed low blood pressure. (b) Attending cardiologist gave adenosine which resulted in change in QRS morphology from wide complex to narrow complex. Underlying rhythm became clear that the child was in atrial fibrillation. The child was then transferred to a paediatric cardiac centre (c) In paediatric high dependency unit, when the child was having IV cannulation, the rhythm switched from atrial fibrillation to a narrow complex AVRT. (d) Oral flecainide was given, and while waiting for adenosine being drawn up the AVRT had resolved  spontaneously and sinus rhythm with overt preexcitation emerged.


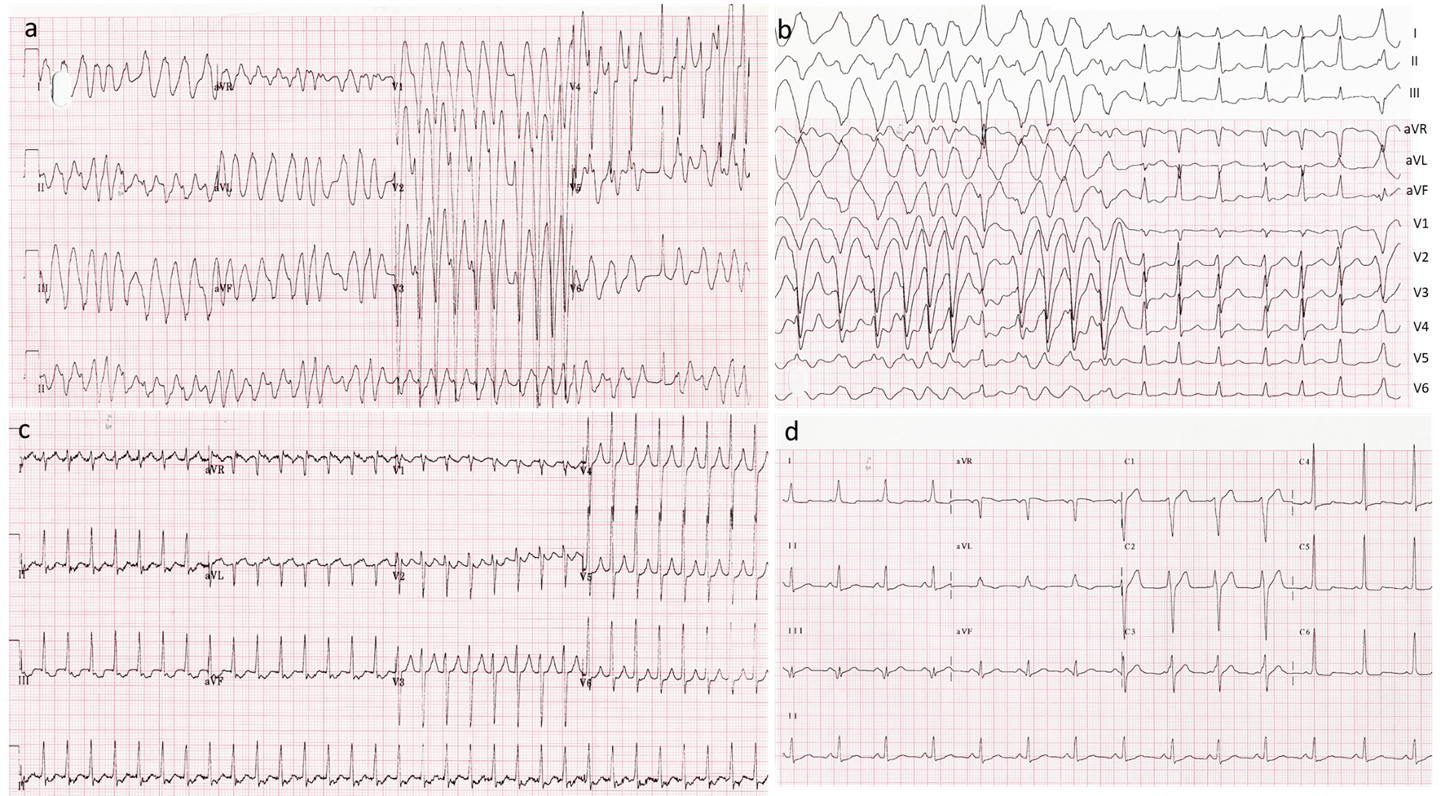

Supplement: Supplementary file 1 — ESM 1 (DOCX 2.21 MB) [file 431_2025_6252_MOESM1_ESM.docx]
