# Supplementary figures and images for: Epidemiology, clinical characteristics and life-threatening risk profile of WPW in children: a single-center experience in South Wales for 30 years
Source: Eur J Pediatr. 2025 Jul 26;184(8):504. doi: 10.1007/s00431-025-06252-z (PMC12296802; doi:10.1007/s00431-025-06252-z)

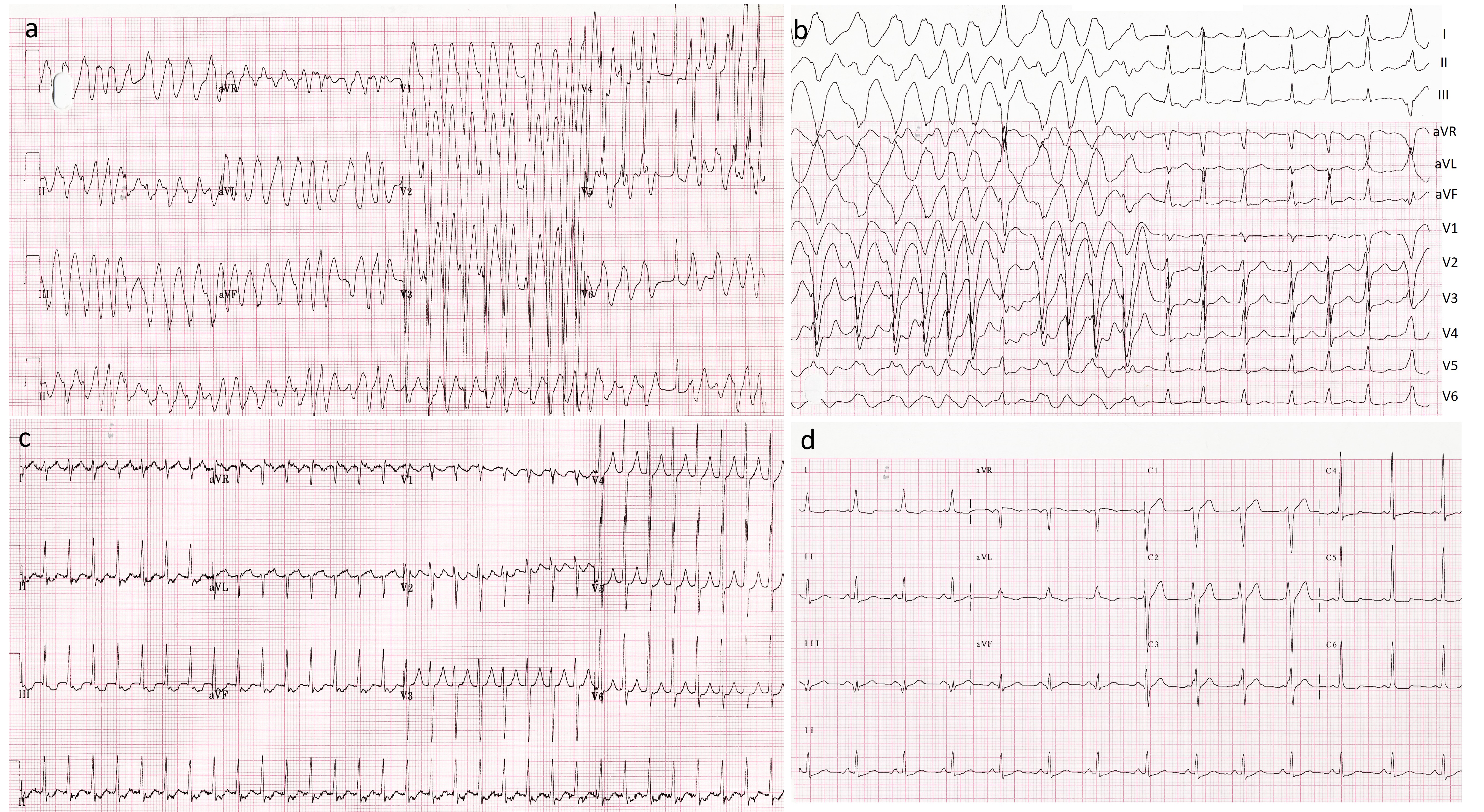

Supplement: Supplementary file 2 — ESM 2 (JPG 4.62 MB) [file 431_2025_6252_MOESM2_ESM.jpg]
